# Air Conditioning Load Data Generation Method Based on DTW Clustering and Physically Constrained TimeGAN

**DOI:** 10.3390/s26010084

**Published:** 2025-12-22

**Authors:** Yu Li, Xiaoyu Yang, Dongli Jia, Wanxing Sheng, Keyan Liu, Rongheng Lin

**Affiliations:** 1 State Key Laboratory of Networking and Switching Technology, Beijing University of Posts and Telecommunications, Beijing 100876, China; rhlin@bupt.edu.cn; 2 China Electric Power Research Institute, Beijing 100892, Chinajiadl@epri.sgcc.com.cn (D.J.);

**Keywords:** time series generation, DTW clustering, TimeGAN, physical constraints, LSTM-based model selection

## Abstract

Generating air-conditioning system load data is crucial for tasks such as power grid scheduling and intelligent energy management. Air-conditioning load data exhibit strong non-stationarity. Their load curves are influenced by seasonal variations and highly correlated with outdoor meteorological conditions, indoor activity patterns, and equipment operational strategies. These characteristics lead to pronounced periodicity, sudden shifts, and diverse data patterns. Existing load generation models tend to produce averaged distributions, which often leads to the loss of specific temporal patterns inherent in air-conditioning loads. Moreover, as purely data-driven models, they lack explicit physical constraints, resulting in generated data with limited physical interpretability. To address these issues, this paper proposes a hybrid generation framework that integrates the DTW clustering algorithm, a physically-constrained TimeGAN model, and an LSTM-based model selection mechanism. Specifically, DTW clustering is first employed to achieve structured data partitioning, thereby enhancing the model’s ability to recognize and model diverse temporal patterns. Subsequently, to overcome the dependency on detailed building parameters and extensive sensor networks, a parameter-free physical constraint mechanism based on intrinsic temperature-load correlations is incorporated into the TimeGAN supervised loss. This design ensures thermodynamic consistency even in sensor-scarce environments where only basic operational data is available. Finally, to address adaptability challenges in long-term sequence generation, an LSTM-based selection mechanism is designed to evaluate and select from clustered submodels dynamically. This approach facilitates adaptive temporal fusion within the generation strategy. Extensive experiments on air-conditioning load datasets from Southeast China demonstrate that the framework achieves a local similarity score of 0.98, outperforming the state-of-the-art model and the original TimeGAN by 11.4% and 13.3%, respectively.

## 1. Introduction

As one of the primary sources of energy consumption in building environments, the air-conditioning (AC) system plays a crucial role in determining overall building energy performance, power system load balance, and energy dispatching efficiency. However, in practical applications, acquiring usable AC load data through traditional physical monitoring faces significant hurdles, including data privacy concerns, sensor faults, high deployment costs, and insufficient historical samples. Therefore, it is of great importance to generate abundant and realistic AC load data using only limited historical information, without relying on extensive building parameters.

Existing time series generation models are mostly based on advanced deep generative architectures, such as Generative Adversarial Networks (GANs), Variational Autoencoders (VAEs), and Denoising Diffusion Probabilistic Models (DDPMs). These models encode complex temporal sequences into a low-dimensional latent space to capture their underlying temporal dependencies and statistical distributions. Although these approaches have demonstrated promising performance in applications such as general sensor signal synthesis and air quality data generation, their direct application to AC load data presents unique challenges. AC loads are highly non-stationary time series whose dynamics are jointly influenced by seasonal cycles, outdoor meteorological conditions (e.g., temperature, humidity, solar radiation), indoor occupant behaviors, and operational control strategies.

During training, GAN-based models are susceptible to mode collapse, which severely restricts the diversity of generated load curves. Consequently, these models struggle to capture load patterns associated with extreme weather conditions or special holidays. VAE-based models, in contrast, tend to generate mean-centered distributions, resulting in overly smooth load curves and the loss of peak–valley details—information critical for grid peak management and demand response strategies. Although DDPMs can achieve high fidelity, their iterative denoising processes are computationally expensive. When trained on limited or noisy datasets, they may fail to learn accurate noise distributions, leading to suboptimal generation quality. Moreover, purely data-driven models often lack explicit physical constraints, whereas thermodynamic laws and strict operational limits inherently govern AC systems. Consequently, data generated by such models may deviate from physical realism, undermining their reliability in applications such as system optimization and fault diagnosis.

To address these limitations, this paper proposes a hybrid model that integrates a DTW-based hierarchical clustering algorithm, a physically-constrained TimeGAN model, and an LSTM-based model selection mechanism. To capture local temporal patterns of AC load data, the proposed framework first applies Dynamic Time Warping (DTW) clustering to segment 96-point load sequences, extracting their major variation trends and representative variance characteristics. Subsequently, a K-Means clustering algorithm is applied to the dimensionally reduced representations, grouping load sequences with similar structures into the same cluster to enable specialized training for each temporal mode.

By integrating physical constraints derived from AC thermodynamics into the TimeGAN supervised loss, the proposed method significantly enhances the physical interpretability and realism of the generated data. Furthermore, considering that different cluster-specific models may exhibit varying adaptability across time segments during long-sequence generation, an LSTM-based model selection mechanism is designed. This mechanism dynamically evaluates the temporal features of samples generated by each cluster-specific TimeGAN submodel. It adaptively selects the most suitable generator at each time step, achieving smooth model transitions and temporal fusion. This design significantly improves the continuity and stability of long-term sequence generation.

Extensive experiments are conducted on AC load datasets collected from diverse building types (including office buildings, commercial complexes, and residential buildings) in Southeast China. The results demonstrate that the proposed hybrid generation framework substantially outperforms conventional generative models across structural diversity, physical plausibility, and temporal consistency.

The main contributions of this paper are summarized as follows:

1. A hybrid generation framework for air-conditioning load data is proposed, which integrates DTW-based clustering into the TimeGAN structure, significantly enhancing the model’s ability to capture local patterns and structural diversity.
2. We introduce a parameter-free physical constraint mechanism that leverages the intrinsic thermodynamic correlation between temperature and load as a regularization term. This innovative design bypasses the need for detailed building envelope parameters, enabling the generation of physically consistent data even in sensor-scarce environments where only basic operational data is available.
3. An LSTM-based model selection mechanism is designed to dynamically schedule and fuse multiple clustered submodels during long-term sequence generation, thereby enhancing continuity and stability.
4. Extensive experiments on real-world AC load datasets show that the proposed model achieves an 11.4% improvement in local data similarity over the current state-of-the-art model and a 13.3% improvement compared to the original TimeGAN.

## 2. Related Work

### 2.1. Time Series Generation Models

Existing time series generation models are primarily based on three architectures: Generative Adversarial Networks (GANs) [1], Variational Autoencoders (VAEs) [2], and Denoising Diffusion Probabilistic Models (DDPMs) [3].

Among GAN-based models, TimeGAN uniquely combines adversarial and supervised learning. It introduces an autoencoder structure to effectively preserve the temporal dependencies and dynamic features of the original time series. By incorporating embedding and supervised losses [4], TimeGAN ensures that the distribution of generated data in the latent space aligns with that of real data, thereby improving data usability. RCGAN (Recurrent Conditional GAN) addresses the problem of conditional time series generation by employing recurrent neural networks as the core of both the generator and discriminator [5]. By conditioning the generation process on label inputs (e.g., categories or contextual info), RCGAN enables precise control over the output sequences. This capability makes it well-suited for multi-category tasks, such as physiological signal synthesis and fault-detection simulation. TTS-CGAN introduces a two-stage generation strategy: the first stage generates coarse-grained time series, and the second stage refines them, improving smoothness and realism [6]. By integrating conditional information, TTS-CGAN enables external control over the generation process, which is particularly advantageous for complex controlled generation tasks.

For VAE-based models, TimeVAE [7] provides a VAE framework tailored for time series modeling, typically combined with recurrent structures (e.g., LSTM [8], GRU [9]) or attention mechanisms for sequence encoding and decoding. The encoder learns latent variable distributions that capture global trends and potential variation patterns, while the decoder reconstructs sequences from these latent representations. In contrast to traditional VAEs, TimeVAE emphasizes temporal dependency modeling, demonstrating superior performance in long-term forecasting and data augmentation tasks. TimeVQ-VAE [10] extends the VAE framework by incorporating a vector quantization mechanism, which uses a discrete latent space to represent more explicit temporal patterns. By encoding sequences into discrete codebook indices and decoding them via lookup operations, TimeVQ-VAE preserves repetitive structures and local patterns more effectively during reconstruction.

TimeDP [11], a DDPM-based model, designs a time series prototype module to extract “prototype vectors” that represent fundamental temporal features. A prototype assignment module then learns domain-specific feature weights to derive domain prompts. During generation, TimeDP can extract conditional prompts from limited samples in a target domain, guiding the diffusion process to produce sequences in the desired style. This design significantly enhances the model’s generalization capability across unseen domains.

Although the above time series generation models can approximate the overall distribution of real data, for domain-specific applications such as air-conditioning load generation, temporal trends, fluctuations, and periodic variations are equally critical. Merely aligning statistical distributions cannot ensure that the generated sequences comprehensively capture the key dynamic characteristics—such as periodicity and non-stationary trends—present in real-world load data.

### 2.2. Clustering-Based Hybrid Models

Clustering-based hybrid models have demonstrated strong potential in various application domains. In time series forecasting, the AW-CNN-LSTM photovoltaic power prediction model developed by the Suzhou Institute of Technical Physics (Chinese Academy of Sciences) employs an elbow-method-based K-means clustering to partition the data [12]. Separate AW-CNN-LSTM models are then trained for each cluster, and their results are adaptively weighted and aggregated to obtain the final forecast. Similarly, researchers from the Electric Power Research Institute of Guizhou Power Grid Co., Ltd. proposed a K-means clustering approach for regional photovoltaic (PV) power stations. Each cluster’s PV power output is predicted individually, and the aggregated results yield more accurate regional PV power forecasts [13].

In other domains, Feras Alasali et al. [14] utilized K-means, hierarchical clustering, and fuzzy C-means algorithms to classify network operating states based on fault current behavior and OCR tripping times. These cluster-based groupings were then combined with bio-inspired optimization techniques—such as the Water Cycle Algorithm (WCA) and the Tunicate Swarm Algorithm (TSA)—to assign optimized time multiplier settings (TMS) to each relay group, ensuring fast, coordinated protection without centralized control or constant communication. Hamed Shams et al. [15] first applied K-means [16] clustering to categorize electricity price, energy demand, wind, and photovoltaic data. These clusters were then integrated into a Particle Swarm Optimization (PSO) framework to determine the optimal capacity and placement of hybrid wind–solar systems, aiming to meet the charging demand of electric vehicle swapping stations.

While clustering methods are ubiquitous in data mining, their integration with time-series generation models remains largely untapped. In particular, there is a lack of systematic research on how to effectively leverage clustering structures to enhance the diversity, realism, and dynamic adaptability of generated time series data.

## 3. Materials and Methods

### 3.1. Model Framework

**Figure 1:**
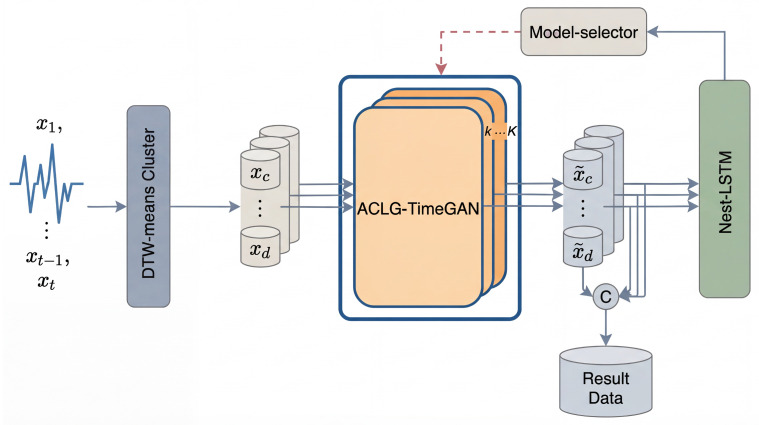
Overall Architecture Diagram.

As shown in Figure 1 and Algorithm 1, this study proposes a generative framework for air-conditioning load time series data that preserves the distributional characteristics, trend information, and fluctuation patterns of the original data. To effectively capture the fluctuation characteristics within the load data, the model first applies a DTW-based hierarchical clustering method to partition the original load sequences into structurally distinct groups. Subsequently, a physically constrained TimeGAN (ACLG-TimeGAN) is trained on each cluster, incorporating air-conditioning system characteristics to enhance physical interpretability. This process yields K representative generative submodels. To address the discontinuity problem commonly encountered during long-term sequence generation and model concatenation, an LSTM-based prediction mechanism is introduced to forecast upcoming temporal patterns. Based on the predicted future state, the model dynamically selects the most suitable ACLG-TimeGAN submodel, thereby improving the continuity and physical plausibility of the generated sequences during temporal transitions.
Algorithm 1:Training Procedure of ACLG-TimeGAN
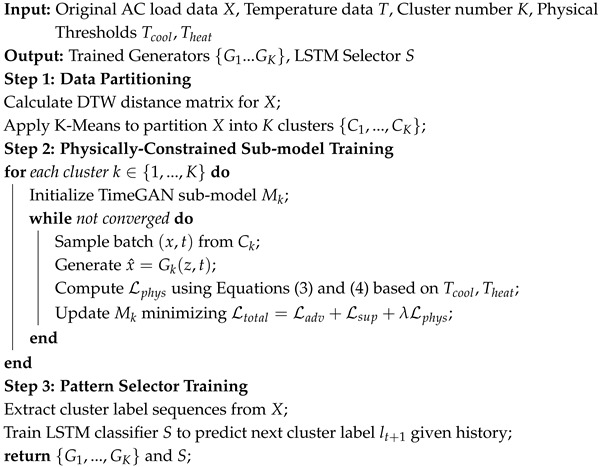


### 3.2. Clustering of Air-Conditioning Load Data

Air-conditioning load profiles are driven by both seasonal temperature variations and abrupt meteorological changes, exhibiting strong non-stationarity and sudden fluctuations. However, the traditional TimeGAN model exhibits inherent limitations in capturing these local fluctuation dynamics due to two primary mechanisms. First, the minimization of the supervised loss (typically Mean Squared Error) forces the model to approximate the conditional expectation of the data distribution; since this average is inherently smoother than individual stochastic realizations, the objective function implicitly filters out high-frequency fluctuations. Second, in the adversarial setting, the discriminator tends to identify sharp variations as outliers, causing the generator to suffer from mode collapse by converging toward the safest, highest-density regions of the distribution—essentially the average behavior—rather than risking the generation of diverse extremes. Consequently, the generated data lacks critical dynamic features such as sudden load spikes, resulting in reduced realism and usability.

(1) $$\begin{array}{cc} L_{U}= & \underset{\mathrm{Loss}\;\mathrm{for}\;\mathrm{real}\;\mathrm{data}}{\underset{︸}{E_{(s,x_{1:T})∼p}\left[\log y_{𝒮}+\sum_{t=1}^{T}\log y_{t}\right]}}\\ \; & +\underset{\mathrm{Loss}\;\mathrm{for}\;\mathrm{generated}\;\mathrm{data}}{\underset{︸}{E_{\left(s,x_{1:T}\right)∼\hat{p}}\left[\log\left(1-{\hat{y}}_{𝒮}\right)+\sum_{t=1}^{T}\log\left(1-{\hat{y}}_{t}\right)\right]}} \end{array}$$

To enhance TimeGAN’s ability to represent the diverse fluctuation patterns of air-conditioning load data—particularly in capturing and reproducing extreme values—a preprocessing strategy based on clustering analysis is employed. Clustering separates different operational states or modes in the dataset (e.g., high/low load periods, startup/shutdown transitions, abnormal operating conditions), allowing each submodel to focus on learning more homogeneous and representative fluctuation patterns and extreme-value distributions.

The quality of clustering is crucial: inappropriate cluster numbers, unsuitable distance metrics, or sensitivity to noise may lead to mixed or uninformative subgroups, introducing bias instead of improvement. Therefore, clustering aims to improve TimeGAN’s recognition and modeling of fluctuation patterns by segmenting load data according to waveform shape similarities.

**Figure 2:**
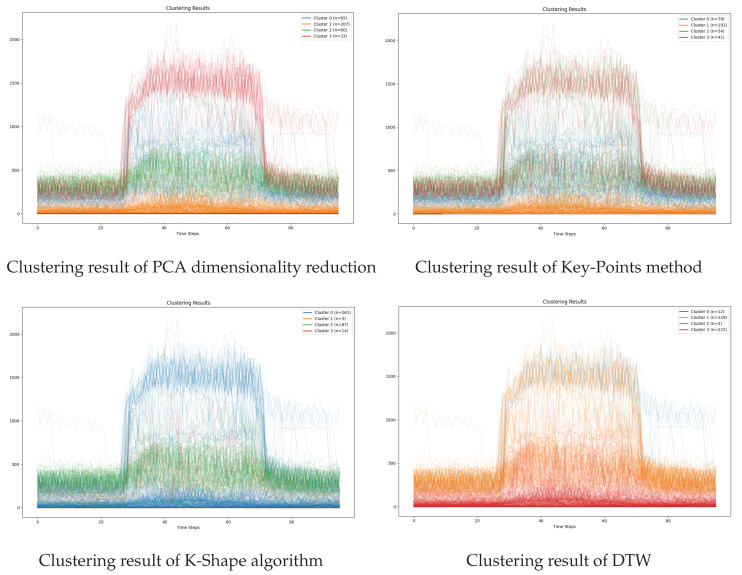
In the DTW-based clustering results, the orange curve represents the “low–high–low” fluctuation pattern, the red curve represents a stable fluctuation pattern, the blue curve represents the “low–high” pattern, and the green curve represents the “high–low” pattern. Compared with other algorithms, the DTW-based clustering results exhibit more distinct fluctuation modes among clusters, clearer hierarchy, and more well-defined cluster boundaries.

As shown in Figure 2. In this study, clustering is performed on 365 days of 96-point air-conditioning load data using four different methods for comparison:

1. **PCA-based clustering [17]:** The load sequences are first reduced in dimensionality via Principal Component Analysis (PCA), followed by K-means clustering in the two-dimensional feature space.
2. **Key-point-based clustering:** Each load curve is represented by a feature vector composed of key points (e.g., extrema, inflection points), and clustering is performed using Euclidean distance-based algorithms.
3. **K-Shape clustering [18]:** The K-Shape algorithm is employed, which measures similarity using Normalized Cross-Correlation (NCC) and clusters standardized load curves directly in the time domain.
4. **DTW-based clustering [19]:** Dynamic Time Warping (DTW) is used to compute pairwise similarity between load sequences, and the resulting distance matrix is used for time series clustering.

### 3.3. ACLG-TimeGAN Subgroup Load Data Generation

The original TimeGAN model, as a general framework for time series generation, is primarily designed to capture the temporal dependencies inherent in sequential data. By integrating the advantages of autoencoding, adversarial training, and supervised learning, TimeGAN effectively learns and reproduces the temporal correlations of the training data. For air-conditioning load data, the model generates time series samples that closely resemble real data, preserving key features such as peak–valley structures and fluctuation patterns. However, the model remains purely data-driven, relying solely on the statistical properties of observed samples. This approach neglects the integration of domain-specific physical knowledge, which is essential for systems governed by complex thermodynamic processes such as air-conditioning systems.

**Figure 3:**
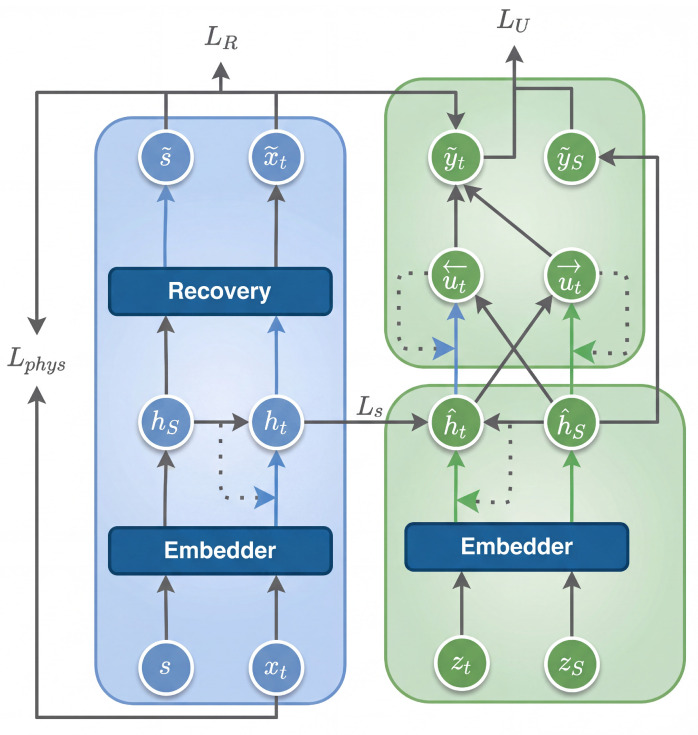
ACLG-TimeGAN.

As a result, the load data generated by the original TimeGAN may violate basic physical laws. For example, simulated indoor temperature variations may fail to align with the input load, outdoor temperature, or building thermal characteristics, thereby violating the principle of energy conservation. As shown in Figure 3. To address this limitation, a physical constraint term is introduced to penalize generated samples that violate thermodynamic principles. This enables the generator to capture not only the data distribution but also fundamental thermodynamic principles, effectively integrating them as prior knowledge.

(2) $$\begin{array}{c} L_{G}^{\mathrm{total}}=L_{U_{G}}+\eta L_{S}+\lambda_{\mathrm{phys}}L_{\mathrm{phys}} \end{array}$$

In practical applications, building-related parameters (e.g., envelope characteristics or HVAC configuration details) are often difficult to obtain. Therefore, the proposed physical constraint is formulated based on the correlation between air-conditioning load variations and temperature dynamics. Specifically, the current operational state of the air-conditioning system cooling or heating is determined according to the temperature trend. Two distinct regions are defined accordingly: the cooling region and the heating region, each associated with different physical constraint formulations.

1. Cooling Zone: When the outdoor temperature *T* is higher than a predefined cooling threshold $T_{\text{cool}\_\text{threshold}}$ (e.g., 26 °C), the air conditioner is assumed to operate in the **cooling mode**. In this mode, the air-conditioning load *P* should have a **positive correlation** with the ambient temperature *T*. That is, the higher the ambient temperature, the greater the cooling demand required to maintain indoor comfort, and consequently, the higher the load. Therefore, the variation in load ΔP should be in the same direction as the variation in temperature ΔT. We penalize cases where the two change in opposite directions. We define the loss function to implement a **selective penalty mechanism:**
  (3) $$\begin{array}{c} L_{\mathrm{cool}}=\mathrm{ReLU}\left(-\Delta P_{\mathrm{gen}}\cdot \Delta T\right) \end{array}$$
  This formulation ensures that physically valid behaviors remain unpenalized. Specifically, if the load and temperature change in the same direction (e.g., a simultaneous decrease, which is physically consistent), their product $\Delta P_{\mathrm{gen}}\cdot \Delta T$ is positive. The negative sign in the formula converts this to a negative input for the ReLU function, resulting in zero loss. The penalty is triggered strictly when the variables exhibit contradictory trends (i.e., opposite signs), which yields a positive input to the activation function.
2. **Heating Zone**: When the ambient temperature *T* is lower than a predefined heating threshold $T_{\mathrm{heat}\_\mathrm{threshold}}$ (e.g., 18 °C), the air conditioner is assumed to operate in the **heating mode**. In this mode, the system follows a **negative correlation**, meaning the load should increase as the temperature drops.
  The loss function is formulated as:
  (4) $$\begin{array}{c} L_{\mathrm{heat}}=\mathrm{ReLU}\left(\Delta P_{\mathrm{gen}}\cdot \Delta T\right) \end{array}$$
  Similarly, this function selectively penalizes only physically implausible scenarios. In valid heating cases where variations are in opposite directions (one increases, the other decreases), their product is negative, causing the ReLU to output zero loss. The loss is activated only when both variables change in the same direction (physically invalid for heating), thereby forcing the model to adhere to thermodynamic principles.

### 3.4. Composite Data Generation

Clustering the AC load data prior to TimeGAN training enables the model to capture distinct fluctuation patterns and enhances sample diversity. However, this cluster-based segmented generation strategy also introduces a notable challenge: the generated temporal segments may become discontinuous and lack overall coherence. Specifically, when TimeGAN models are trained independently to generate daily load curves corresponding to different operational types or load levels, the transition boundaries between consecutive sequences are often neglected. Consequently, transitions between ’weekday’ and ’weekend’ sequences may exhibit abrupt jumps or unnatural discontinuities, deviating from the smooth, continuous variations typical of real-world load data.

To mitigate boundary discontinuities, this study introduces an LSTM-guided sequence splicing mechanism that adaptively determines the generation order and ensures smooth transitions. Specifically, the process operates as follows. First, a set of pre-trained TimeGAN models $M_{GAN}$ is used, where each model corresponds to a distinct load pattern cluster (e.g., low–high–low, stable, high–low, etc.). In parallel, an LSTM-based pattern prediction model $M_{LSTM}$ is trained to learn the temporal evolution of cluster modes from the historical sequence.

During the generation phase, the procedure starts from an initially generated sequence $S_{Current}$. At each iteration, the recent historical portion of $S_{Current}$ is extracted and fed into $M_{LSTM}$ to predict the probability distribution of the next most likely load pattern. The pattern with the highest predicted probability, denoted as next_mode, is then selected. Subsequently, the corresponding TimeGAN model $M_{GAN}\left[next\_mode\right]$ generates a new one-day synthetic load sequence $S_{new\_day}$. After generation, $S_{new\_day}$ is concatenated with the existing sequence $S_{Current}$ through a splicing and smoothing operation, which aligns the boundary values and applies local smoothing to ensure temporal continuity between adjacent days. This iterative process continues until the desired length of the synthetic time series is achieved.

Through this LSTM-guided approach, the model not only maintains diversity among different load modes but also ensures that the transition between consecutive generated segments remains natural and coherent, thereby restoring the smooth day-to-day variation characteristics observed in real-world load data.

## 4. Experiment and Analysis

### 4.1. Dataset

**Data Info:** The experiments in this study are conducted on the air-conditioning load dataset collected from an administrative office building located in southeastern China. The dataset covers the entire year of 2023, comprising 96 data points per day that record the central air-conditioning load. In total, it contains 35,040 time-series samples (365 days × 96 samples/day). This dataset effectively captures the operational characteristics and fluctuation patterns of public building air-conditioning systems across different seasons and time periods, making it a representative benchmark for real-world load generation and modeling.

**Data Preprocessing:** To ensure data quality, we first addressed missing values, which comprised approximately 0.5% of the raw dataset. Short gaps (<2 h) were filled using linear interpolation, while days with longer continuous missing periods were excluded. Outliers caused by sensor errors were detected using the 3σ principle and replaced with the moving average of adjacent points.

### 4.2. Baseline Models and Evaluation Metrics

To assess the proposed model in air-conditioning load generation, we benchmark it against state-of-the-art models based on GAN, VAE, and DDPM frameworks.

1. TimeGAN: TimeGAN combines adversarial training with supervised learning, enabling high-fidelity sequence generation while preserving temporal dependencies. As a core component of the proposed hybrid model, it serves as an essential baseline to validate the effectiveness of integrating clustering and LSTM-based composition modules.
2. TimeVAE and TimeVQVAE: TimeVAE is a VAE-based time-series generative model that utilizes continuous latent variables to reconstruct and generate diverse temporal samples. TimeVQVAE extends this framework by incorporating vector quantization, learning discrete latent representations that better capture local structural information and higher-order temporal dependencies. Comparing our model with TimeVAE and TimeVQVAE helps assess its capability to maintain diversity, fidelity, and distributional consistency, particularly in representing complex latent dynamics.
3. TimeDP: TimeDP leverages a diffusion probabilistic model for time-series generation and has demonstrated remarkable success in recent years, especially in modeling complex distributions and achieving stable generation. Using TimeDP as a benchmark highlights our method’s advantages in high-quality generation and accurate reconstruction of complex temporal dynamics.

To systematically evaluate the quality of the generated data, we perform a comparative analysis from the following three perspectives:

1. Comprehensiveness: The generated samples should adequately cover the distribution of real data. We employ t-distributed Stochastic Neighbor Embedding (t-SNE) to project both real and generated samples into a two-dimensional feature space. The degree of overlap and similarity in the resulting visualization reflects the model’s ability to capture the overall data structure and ensure distributional diversity.
2. Similarity: Beyond overall coverage, the generated data should exhibit strong consistency with the real data in terms of statistical properties and temporal dynamics. Accordingly, we adopt Dynamic Time Warping (DTW), Time-series Similarity (TCS), and the Kolmogorov–Smirnov (KS) test to quantify discrepancies between generated and real samples. These metrics comprehensively evaluate the model’s capacity to reproduce realistic fluctuation patterns, amplitude variations, and dynamic trends.
3. Availability: The generated data should possess practical utility for downstream applications. To evaluate data utility, we train a time-series forecasting model (e.g., an LSTM) on synthetic air-conditioning load data and assess its performance on a real-world test set. Suppose the model trained on synthetic data achieves results comparable to or better than those trained on real data. In that case, it demonstrates the high practical utility and fidelity of the generated data.

### 4.3. Results

**Figure 4:**
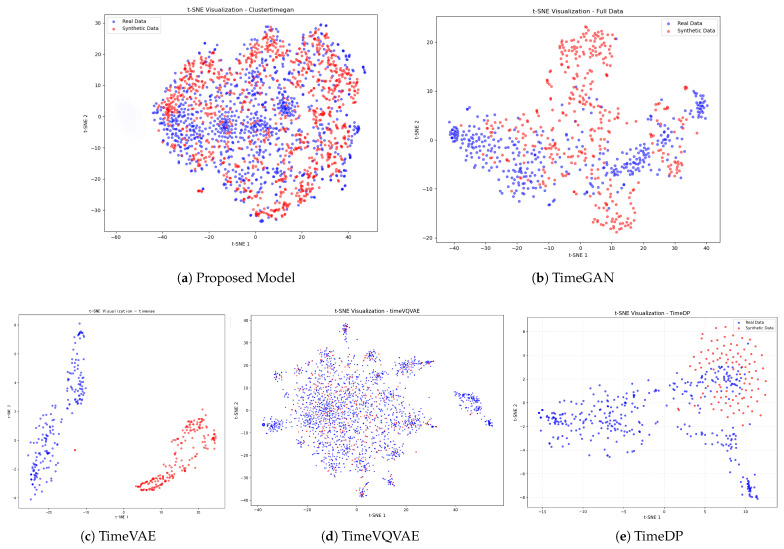
Red points represent real data, while blue points represent generated data. A higher degree of overlap indicates better local consistency between the generated and real samples.

**Comprehensiveness:** In terms of data comprehensiveness, this study selects portions of the original and generated load data from commercial buildings for t-SNE analysis (with red dots representing original data and blue dots representing generated data) [20]. As shown in Figure 4, compared with other baseline models, the data generated by the proposed model more comprehensively cover the distribution range of the original data. This fully demonstrates that the proposed model achieves superior comprehensiveness in representing the diversity of real-world data.

**Similarity:** Table 1 presents the similarity evaluation results of the proposed model and other baseline models on the commercial building air-conditioning load dataset. The proposed model consistently outperforms the comparison models across all metrics. Specifically, for the TCS metric (reflecting local similarity), the proposed model achieves a score of **0.98**, outperforming the best baseline, TimeVQVAE (**0.88**), by approximately **11.4%**. This demonstrates a superior capability in capturing local features and validates the effectiveness of the clustering module in the data generation process. For the DTW, MMD, and KS metrics, which measure global distribution similarity, the proposed model achieves improvements of approximately **31.8%**, **25.0%**, and **16.0%** over TimeVQVAE, respectively. These results highlight the model’s superiority in global distribution modeling. Overall, the proposed model achieves substantial gains in local and global similarity metrics. This confirms that combining the LSTM architecture with a clustering-based mechanism significantly enhances the model’s capacity to learn intrinsic data patterns.

**Efficiency:** The results demonstrate that the proposed model achieves an optimal balance between generation quality and computational efficiency. While TimeVAE exhibits the fastest execution speeds, its significantly lower fidelity (as indicated by the highest DTW score of 26.23) limits its practical utility. In contrast, our model demonstrates a clear advantage in inference efficiency over the diffusion-based TimeDP. Specifically, our model generates the complete test set approximately 5.5 times faster (10.55 s) than TimeDP (58.20 s), effectively mitigating the computational latency typically associated with the iterative denoising process in diffusion models. Furthermore, although the training time is marginally higher than the original TimeGAN due to the integration of physical constraints and the LSTM module, this slight increase is negligible compared to the substantial gains in accuracy (e.g., a 49.4% reduction in DTW compared to TimeGAN), confirming the model’s suitability for practical, high-fidelity data generation.

**Availability:** In the experiments where the generated data were used to train an LSTM prediction model, the predictive performance obtained using data generated by the proposed model consistently surpassed that of other comparison models. Specifically, on the office building air-conditioning load dataset, the prediction model trained with the proposed model’s generated data achieved **42.42%** and **44.00%** lower Root Mean Square Error (RMSE) and Mean Absolute Error (MAE), respectively, than those trained with TimeGAN data. Compared with TimeDP, the reductions were **45.71%** and **51.72%**, while the RMSE and MAE were also **9.52%** and **12.50%** lower than those of TimeVQVAE. These results fully demonstrate that the data generated by the proposed model effectively preserves the key features and latent patterns of real data, exhibiting high practical applicability. The generated data can serve as a supplement or substitute for real-world data in model training and system simulation scenarios. Particularly in cases where real data are difficult to obtain or the sample size is insufficient, the proposed model can significantly enhance the performance and generalization capability of downstream tasks.

### 4.4. Ablation Study

**Ablation Study on the Number of Clusters:** To verify the practical role of the clustering module in the proposed model and to analyze the impact of different numbers of clusters on model performance, an ablation experiment was conducted on the office building dataset. The results are shown in Table 2. From the table, it can be observed that when the clustering module is not introduced (K=1), the overall model performance is relatively poor. The model performs worse in both similarity metrics (e.g., DTW, KS, MMD) and availability metrics (e.g., RMSE, MAE, MAPE). For example, DTW is 12.67, MMD is 0.26, and RMSE is 0.33. As the number of clusters *K* increases, the model’s performance gradually improves across all metrics. When the number of clusters reaches K=4, the model achieves the best performance: DTW decreases to 6.41 (a reduction of approximately 49.4% compared to K=1), MMD decreases to 0.12 (a reduction of about 53.8%), and TCS increases to 0.98 (an improvement of about 15.3%). Meanwhile, RMSE, MAE, and MAPE drop to 0.19, 0.14, and 4.6%, respectively, indicating that the generated data at this point most closely matches the real data in both global distribution and local structure, thus providing the best support for downstream load forecasting tasks. However, when the number of clusters is further increased to K=8 or K=16, the performance metrics deteriorate significantly (e.g., DTW rises to 14.82 and 26.35, while TCS decreases to 0.81 and 0.43). This suggests that excessive clustering reduces the number of samples within each subgroup, thereby weakening the model’s learning effectiveness. In summary, a moderate number of clusters (e.g., K=4) can significantly enhance the model’s ability to capture both local features and global structural distributions, thereby improving the quality of generated data and its applicability in downstream tasks. These experimental results fully demonstrate the effectiveness and necessity of the clustering mechanism in the proposed model.

**Ablation Study on the models:** Table 3 presents the ablation study results, quantitatively validating the necessity of each key module. The removal of the physical constraint module leads to a noticeable performance decline (e.g., DTW increases from 6.41 to 8.35), indicating that thermodynamic regularization is essential for preventing physically implausible samples and ensuring high fidelity. Furthermore, removing the LSTM-based combination mechanism results in even more severe degradation, with the DTW score spiking to 10.72. This underscores the critical role of dynamic scheduling in maintaining temporal continuity and smoothing transitions between different load patterns. Ultimately, the Full Model achieves the best performance across all similarity and predictive metrics, confirming that the synergistic integration of physical constraints and LSTM-based fusion is indispensable for generating high-quality air-conditioning load data.

**Ablation Study on the temperature threshold:** Table 4 presents the sensitivity analysis of the cooling and heating temperature thresholds, evaluating their impact on generation quality. The results demonstrate that the proposed setting (26 °C/18 °C) achieves the optimal balance between physical consistency and data diversity. Conversely, overly strict thresholds impose rigid thermodynamic laws on transitional zones dominated by stochastic user behavior, leading to over-regularization.On the other hand, overly loose thresholds (or the absence of physical constraints) fail to provide sufficient guidance, causing the model to regress toward a purely data-driven distribution. This limitation is most evident in the generation of physically implausible samples where the load drifts independently of temperature trends. For example, in a cooling scenario without effective constraints, the model might generate a trajectory where the outdoor temperature rises significantly, yet the air-conditioning load paradoxically decreases or exhibits random fluctuations. This violation of the fundamental positive correlation mandated by thermodynamics further validates the necessity and robustness of the selected parameters.

## 5. Conclusions

To address the challenges of strong non-stationarity, weak physical consistency, and insufficient pattern coverage in air-conditioning load data generation tasks, this paper proposes an improved TimeGAN model that integrates DTW-KMeans clustering, physical constraints, and an LSTM-based dynamic scheduling mechanism. By analyzing the fluctuation structure of air-conditioning load data, the proposed approach employs a clustering-guided mechanism to train sub-models, effectively enhancing the capture of diverse load patterns. By incorporating physical characteristics of air-conditioning systems as constraints, the method effectively improves the physical plausibility of the generated data. Furthermore, by integrating an LSTM-based prediction mechanism, the model enables dynamic selection and seamless concatenation of sub-models, thereby significantly improving the continuity and consistency of the synthetic data.

Addressing scenarios with sensor scarcity where only temperature and load data are available, the proposed method leverages a thermodynamic physical constraint mechanism to generate high-fidelity, physically consistent data without requiring additional building parameters, thereby effectively overcoming the data generation bottleneck associated with low-dimensional monitoring.

Experimental results on real-world air-conditioning load datasets from Southeast China demonstrate that the proposed model outperforms existing mainstream time-series generation models in terms of comprehensiveness, similarity, and availability. The t-SNE visualizations and multiple statistical indicators further confirm its superior ability to reconstruct structural patterns and capture dynamic features. Moreover, the model demonstrates superior robustness and accuracy in downstream load forecasting. Crucially, the practical utility of this work extends beyond prediction; such high-fidelity synthetic data can serve as a critical input for training and validating advanced energy management algorithms, including model predictive control or reinforcement learning agents that require extensive and diverse operational scenarios. Ablation studies further validate the positive contributions of the clustering, physical constraint, and LSTM modules to the quality of generated data.

## Figures and Tables

**Table 1:** Performance and Efficiency Comparison of Generative Models. (Bold indicates the best performance).

Model	Similarity Metrics				Predictive Error Metrics			Efficiency Metrics	
	DTW↓	TCS↑	KS↓	MMD↓	RMSE↓	MAE↓	MAPE↓	Train (s/ep)↓	Infer (s)↓
TimeGAN	12.67	0.85	0.24	0.26	0.33	0.25	5.0%	12.5	9.42
TimeVAE	26.23	0.32	0.50	0.39	0.31	0.25	5.2%	**8.2**	**5.35**
TimeVQVAE	9.40	0.88	0.25	0.16	0.21	0.16	4.8%	14.1	7.48
TimeDP	18.25	0.68	0.35	0.21	0.35	0.29	5.3%	45.3	58.20
**Proposed**	**6.41**	**0.98**	**0.21**	**0.12**	**0.19**	**0.14**	**4.6%**	14.2	10.55

Note: “Train (s/ep)” denotes the average training time per epoch in seconds. “Infer (s)” denotes the total time required to generate the complete test set (35,040 samples).

**Table 2:** Impact of Different Numbers of Clusters (*K*) on Model Performance. (Bold indicates the best performance).

Clusters (*K*)	Similarity Metrics				Predictive Error Metrics		
	DTW↓	TCS↑	KS↓	MMD↓	RMSE↓	MAE↓	MAPE↓
1	12.67	0.85	0.24	0.26	0.33	0.25	5.0%
2	8.54	0.91	0.22	0.19	0.27	0.21	4.8%
**4**	**6.41**	**0.98**	**0.21**	**0.12**	**0.19**	**0.14**	**4.6%**
8	14.82	0.81	0.26	0.28	0.35	0.27	5.2%
16	26.35	0.43	0.51	0.35	0.37	0.29	5.6%

**Table 3:** Ablation Study Results of Key Modules. (Bold indicates the best performance).

Model Variant	Similarity Metrics				Predictive Error Metrics		
	DTW↓	TCS↑	KS↓	MMD↓	RMSE↓	MAE↓	MAPE↓
**Proposed (Full Model)**	**6.41**	**0.98**	**0.21**	**0.12**	**0.19**	**0.14**	**4.6%**
w/o Physical Constraint	8.35	0.93	0.23	0.18	0.24	0.18	4.9%
w/o LSTM Combination	10.72	0.90	0.25	0.23	0.27	0.21	5.1%

**Table 4:** Sensitivity Analysis of Temperature Thresholds ($T_{cool}$/$T_{heat}$). (Bold indicates the best performance).

Threshold Settings	Similarity Metrics				Predictive Error Metrics		
	DTW↓	TCS↑	KS↓	MMD↓	RMSE↓	MAE↓	MAPE↓
$T_{cool}=28\;{}^{\circ}C,T_{heat}=16\;{}^{\circ}C$	7.82	0.94	0.22	0.16	0.22	0.17	4.8%
$T_{cool}=24\;{}^{\circ}C,T_{heat}=20\;{}^{\circ}C$	6.85	0.96	0.21	0.14	0.20	0.15	4.7%
** Proposed (26 °C/18 °C)**	**6.41**	**0.98**	**0.21**	**0.12**	**0.19**	**0.14**	**4.6%**

## Data Availability

The original contributions presented in this study are included in the article. Further inquiries can be directed to the corresponding authors.
